# A novel *AFG3L2* mutation close to AAA domain leads to aberrant OMA1 and OPA1 processing in a family with optic atrophy

**DOI:** 10.1186/s40478-020-00975-w

**Published:** 2020-06-29

**Authors:** Valentina Baderna, Joshua Schultz, Lisa S. Kearns, Michael Fahey, Bryony A. Thompson, Jonathan B. Ruddle, Aamira Huq, Francesca Maltecca

**Affiliations:** 1 Mitochondrial dysfunctions in neurodegeneration Unit, Division of Neuroscience, Ospedale San Raffaele, Milan, Italy; 2 Parkville Familial Cancer and Genomic Medicine Department, The Royal Melbourne Hospital, Parkville, Australia; 3 Centre for Eye Research, East Melbourne, Australia; 4 Royal Victorian Eye and Ear Hospital, East Melbourne, Australia; 5 Department of Pathology, The Royal Melbourne Hospital, Parkville, Australia; 6 Department of Medicine, University of Melbourne, Parkville, Australia; 7 Università Vita-Salute San Raffaele, Milan, Italy

**Keywords:** AFG3L2, OPA1, Optic atrophy, Mitochondrial fragmentation

## Abstract

Autosomal dominant optic atrophy (ADOA) is a neuro-ophthalmic condition characterized by bilateral degeneration of the optic nerves. Although heterozygous mutations in *OPA1* represent the most common genetic cause of ADOA, a significant number of cases remain undiagnosed.

Here, we describe a family with a strong ADOA history with most family members spanning three generation having childhood onset of visual symptoms. The proband, in addition to optic atrophy, had neurological symptoms consistent with relapsing remitting multiple sclerosis. Clinical exome analysis detected a novel mutation in the *AFG3L2* gene (NM_006796.2:c.1010G > A; p.G337E), which segregated with optic atrophy in family members. AFG3L2 is a metalloprotease of the AAA subfamily which exerts quality control in the inner mitochondrial membrane. Interestingly, the identified mutation localizes close to the AAA domain of AFG3L2, while those localized in the proteolytic domain cause dominant spinocerebellar ataxia type 28 (SCA28) or recessive spastic ataxia with epilepsy (SPAX5)*.* Functional studies in patient fibroblasts demonstrate that the p.G337E AFG3L2 mutation strongly destabilizes the long isoforms of OPA1 via OMA hyper-activation and leads to mitochondrial fragmentation, thus explaining the family phenotype. This study widens the clinical spectrum of neurodegenerative diseases caused by *AFG3L2* mutations, which shall be considered as genetic cause of ADOA.

## Introduction

ADOA is a genetic condition affecting the retinal ganglion cells (RGCs), whose axons form the optic nerve. It is a relatively common form of inherited optic neuropathy, with a prevalence of 3/100,000 in most populations worldwide. Patients are usually diagnosed during early childhood, because of bilateral visual loss related to optic disc pallor or atrophy, and typically in the context of a family history of ADOA.

Molecular diagnosis is provided by the identification of a mutation in the *OPA1* gene (75% of ADOA patients) or in the *OPA3* gene (1% of patients) [1]. However, many ADOA cases remain undiagnosed [2].

About 20% of patients with *OPA1* mutations are known to develop additional co-morbidities of deafness, ophthalmoplegia, ataxia, myopathy and peripheral neuropathy [1]*.*

OPA1 is a mitochondrial GTPase responsible for the fusion of the inner mitochondrial membrane (IMM), by which it regulates mitochondrial dynamics. OPA1 is transcribed in eight splicing isoforms which undergo constitutive processing in the IMM. At steady state, OMA1-operated cleavage at S1 site and/or YME1L1-operated cleavage at S2 site lead to the generation of long- (non-cleaved) and short- (cleaved) forms of OPA1 [3]. OPA1 long forms (L-OPA1) are the active mediators of mitochondrial fusion, and their processing to short, soluble forms (S-OPA1) limits fusion and can facilitate mitochondrial fragmentation [4, 5].

OPA1 processing is finely regulated by AFG3L2, a mitochondrial protein belonging to the AAA-protease subfamily (ATPases associated with various cellular activities) which exerts quality control in the IMM [6]. We previously demonstrated that loss of AFG3L2 induces hyper-activation of the stress-activated protease OMA1 and leads to excessive OPA1 processing, promoting mitochondrial fragmentation [7]. Mutations in the proteolytic domain of AFG3L2 cause dominant SCA28 and the rare recessive SPAX5, whose main clinical features are gait ataxia and lack of balance with cerebellar atrophy [8, 9].

In this report, we describe a family with ADOA. By clinical exome analysis we identified a novel mutation in AFG3L2 (p.G337E) segregating with optic atrophy within the family. In contrast to SCA28 causing-mutations, which mostly affect the proteolytic domain, this new mutation localizes close to the AAA domain of AFG3L2. Functional studies demonstrate that the p.G337E mutation abolishes AFG3L2 function and leads to a striking L-OPA1 destabilization, comparable to the one observed in *Afg3l2* null cells. Our data disclose OMA1 hyper-activation, OPA1 enhanced processing and mitochondrial fragmentation as the pathogenic cascade of ADOA caused by AFG3L2 p.G337E mutation.

## Case presentation

**Fig. 1:**
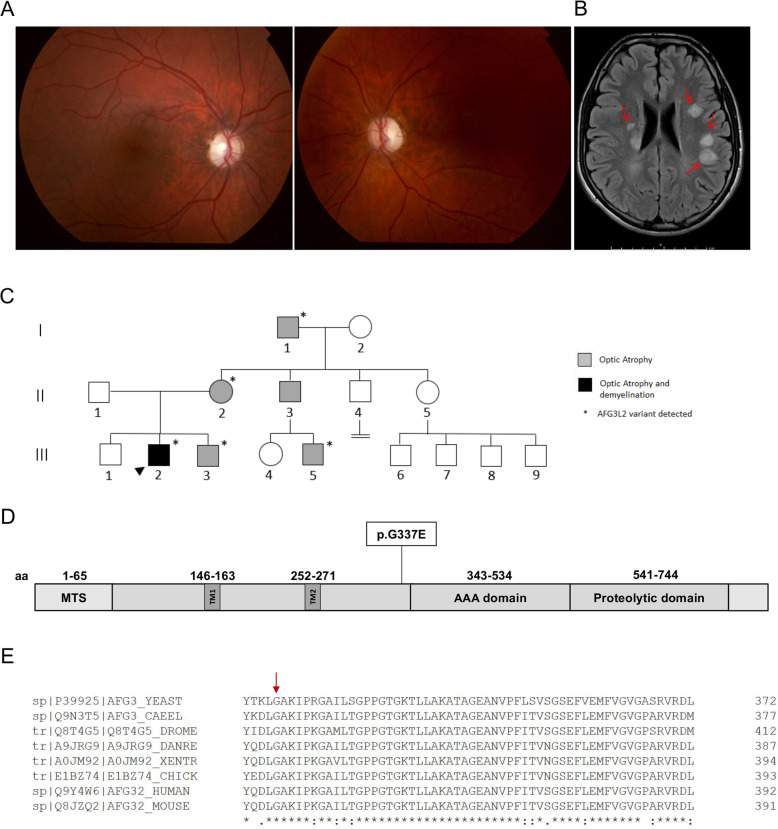
Family clinical features and pedigree. **a** Fundus photos of proband age 20 showing bilateral optic nerve atrophy. **b** MRI Brain demonstrating numerous T2/FLAIR hyperintense lesions predominantly involving the periventricular white matter and the grey-white matter junction. **c** Pedigree demonstrating clear autosomal dominant inheritance of optic atrophy. The arrow indicates the proband. **d** AFG3L2 protein scheme with functional domains, reporting the mutation described here. **e** p.G337 AFG3L2 residue conservation among different AFG3L2 orthologues

The proband was diagnosed with optic atrophy aged 4, when he was found to have reduced vision (right 3/60, left 2/60), poor color perception with Ishihara testing and mild optic atrophy. Electrophysiology investigation revealed poor amplitudes with visual evoked potentials and a normal electroretinogram. Brain Magnetic Resonance Imaging (MRI) at the age of 5 was normal. Optic atrophy slowly worsened with age, showing marked optic nerve pallor aged 20 (Fig. 1a). The proband also presented with an acute episode of cerebellar ataxia at the age of 18 and was diagnosed with relapsing remitting multiple sclerosis (MS). He fulfilled the McDonald criteria for diagnosis of MS and brain MRI demonstrated widespread demyelinating lesions in both cerebral, cerebellar hemispheres as well as the midbrain and cord (Fig. 1b). His cerebrospinal fluid (CSF) analysis showed oligoclonal bands. Anti-aquaporin 4 antibodies (Neuromyelitis optica-Immunoglobulin G - NMO IgG) testing was negative. His symptoms improved after plasma exchange and he is now stable on monthly Natalizumab infusions. There was a known history of mild ADOA in this family, with proband’s mother, maternal grandfather and multiple other maternal relatives being affected by optic atrophy but able to drive, with vision of at least 6/12. The proband’s younger brother was found to have a similar severe level of vision and optic atrophy aged 5 (Fig. 1c). None of the family members had symptoms of spinocerebellar ataxia.

### Genetic testing

We identified a heterozygous missense mutation NM_006796.2(AFG3L2):c.1010G > A in exon 8 of the *AFG3L2* gene in a family member with optic atrophy. This is a novel mutation, not reported in population databases such as gnomAD or in clinical cases, resulting in glycine to glutamic acid position 337 NP_006787.2(AFG3L2):p.G337E. This mutation segregates with optic atrophy in five family members in total and followed an autosomal dominant pattern of inheritance (Fig. 1c and d). p.G337E is very highly conserved and in silico softwares consistently predict it to be pathogenic (Fig. 1e).

### Functional studies

**Fig. 2:**
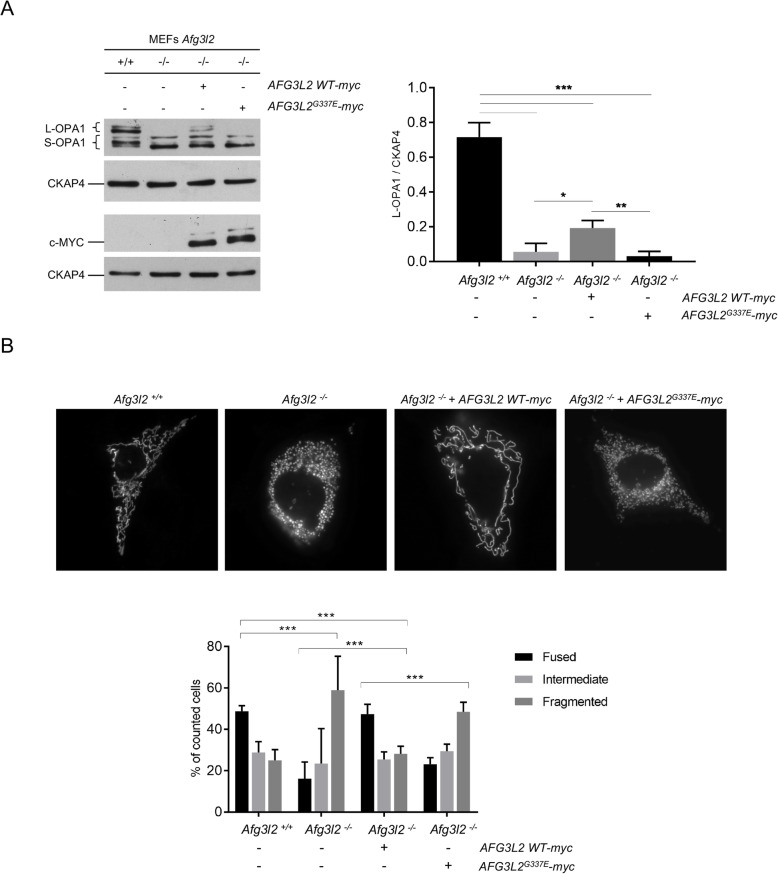
Overexpression of exogenous *AFG3L2*^*G337E*^-myc in a AFG3L2 null background does not rescue L-OPA1 and mitochondrial tubulation. **a** WB analysis and relative quantification of L-OPA1 after transient transfection of mt-YFP in combination with *AFG3L2 WT-myc* or *AFG3L2*^*G337E*^*-myc* in *Afg3l2*^*+/+*^ and *Afg3l2*^*−/−*^ MEFs (ratio 1:3). c-MYC was used as transfection control. Bars represent means ± SEM of three independent experiments. Student’s t test: * *p* < 0.05; ** *p* < 0.01; *** *p* < 0.001. **b** Representative pictures of in live mitochondrial morphology after transient transfection of mt-YFP in combination with *AFG3L2 WT-myc* or *AFG3L2*^*G337E*^*-myc* in *Afg3l2*^*+/+*^ and *Afg3l2*^*−/−*^ MEFs (ratio 1:3). The graph shows the morphometric analysis of mitochondrial morphology. At least 80 randomly selected cells were analyzed in each experiment. Chi-square analysis (two degrees of freedom): *** p < 0.001

To functionally assay the pathogenicity of the p.G337E mutation, we mutagenized an *AFG3L2 WT*-*myc* construct to obtain *AFG3L2*^*G337E*^*-myc* and overexpressed it in *Afg3l2*^−/−^ MEFs. We analyzed by WB the levels and post translational processing of OPA1. In *Afg3l2*^−/−^ MEFs OPA1 is processed at a higher rate compared to wt controls, leading to a striking reduction of L-OPA1, which inhibits fusion and triggers mitochondrial network fragmentation [7, 10]. We found that overexpression of *AFG3L2*^*G337E*^*-myc* does not restore, even partially, L-OPA1 in *Afg3l2*^−/−^ MEFs. Conversely, L-OPA1 are recovered by *AFG3L2 WT-myc* overexpression, indicating that the p.G337E mutation completely abolishes AFG3L2 activity (Fig. 2a).

We then analyzed mitochondrial morphology in *Afg3l2*^−/−^ MEFs transfected with mt-YFP alone, or in combination with *AFG3L2*^*G337E*^*-myc* or *AFG3L2 WT-myc.* Strikingly, and in accordance with L-OPA1 enhanced processing, the mitochondrial network was comparable between *Afg3l2*^−/−^ MEFs and *Afg3l2*^−/−^ MEFs transfected with *AFG3L2*^*G337E*^*-myc,* with the highest percentage of cells showing fragmented mitochondria. Conversely, the overexpression of *AFG3L2 WT-myc* was able to restore mitochondrial tubulation in *Afg3l2*^−/−^ MEFs (Fig. 2b).

**Fig. 3:**
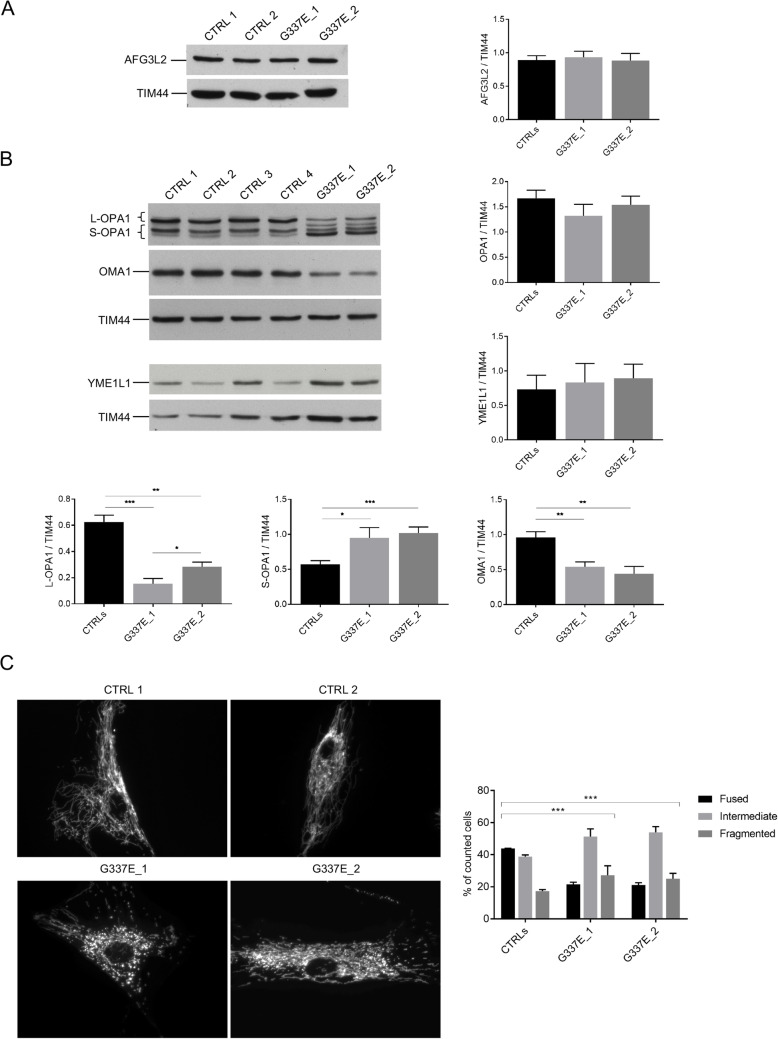
Patient fibroblasts show enhanced L-OPA1 processing via OMA1 hyper-activation. WB analysis and relative quantification of (**a**) AFG3L2 and (**b**) OPA1 total amount, YME1L1, L-OPA1, S-OPA1, OMA1 in human primary fibroblasts. G337E_1 is the proband and G337E_2 is patient II-2 of the pedigree. Bars represent means ± SEM of three independent experiments. Student’s t test: * p < 0.05; ** p < 0.01; *** p < 0.001. (**c**) Representative pictures of mitochondrial morphology in human primary fibroblasts infected with mtDsRed2 and visualized by live imaging microscopy. The graph shows the morphometric analysis of mitochondrial morphology. At least 100 randomly selected cells were analyzed in each experiment. Chi-square analysis (two degrees of freedom): *** p < 0.001

We then evaluated the effect of the p.G337E mutation in a more physiopathologic context, by analyzing primary fibroblasts from the proband (III-2) and his affected mother (II-2). We firstly assayed by WB the effect of the p.G337E mutation on the stability of AFG3L2 monomer and we found that the whole amount of AFG3L2 is comparable between patient and control fibroblasts (Fig. 3a). On the contrary, we observed that L-OPA1 are strongly destabilized by an enhanced processing in patient fibroblasts compared to controls, leading to a striking reduction of L-OPA1 and to an accumulation of S-OPA1 (Fig. 3b). We also proved that this is due to the hyper-activation of OMA1, as demonstrated by a significant reduction in its amount when compared to controls (Fig. 3b). Indeed, we previously showed that OMA1 is hyper-activated in the absence of AFG3L2 and undergoes autocatalytic degradation [7, 11]. We also evaluated possible compensatory effects of YME1L1, but the levels of this protease resulted comparable between patients and controls (Fig. 3b).

Live imaging evaluation of mitochondrial network showed a highly fused and interconnected mitochondria in control cells, while patient fibroblasts carrying the p.G337E mutation presented shorter and more isolated organelles, consistent with OPA1 abnormal processing (Fig. 3c).

## Discussion and conclusions

Here we describe a family in which a novel heterozygous mutation in *AFG3L2* co-segregates with ADOA. Interestingly, the identified p.G337E mutation localizes close to the AAA domain of AFG3L2, in contrast with those causing SCA28 and SPAX5, which clusterize in the proteolytic domain of the protein. Functional studies of the p.G337E mutation demonstrate its pathogenicity, as it strongly affects the processing of L-OPA1 leading to aberrant mitochondrial fragmentation. The defective mitochondrial dynamics is in line with most optic neuropathies exhibiting mitochondrial dysfunction in RGCs as underlying mechanism [12].

Other heterozygous/compound heterozygous mutations in *AFG3L2* have been described in isolated cases with nonsyndromic optic atrophy (although not supported by functional studies) [13, 14], and in familial syndromic and non-syndromic optic atrophy [15, 16]. The present study further demonstrates that heterozygous mutations in *AFG3L2* shall be considered as a genetic cause for ADOA in *OPA1* and *OPA3*- negative cases.

The family described here has features of ADOA with no clinical evidence of spinocerebellar ataxia in any of the affected members but the proband, who experienced an acute episode of cerebellar ataxia. As proband’s clinical features, MRI and CSF investigations were consistent with relapsing remitting MS and the demyelinating lesions (including the cerebellar lesions) settled on treatment with Natalizumab, his cerebellar symptoms were most likely due to MS. However, it remains to be determined if these demyelinating neurological symptoms could be directly related to the AFG3L2 mutation. Although mouse studies have shown that deletion of AFG3L2 (either constitutive or in mature mouse oligodendrocytes) can cause myelin abnormalities [17, 18], no such phenotype has been described in humans before. Comorbidity cannot therefore be excluded in the proband.

The functional studies we conducted on the p.G337E mutation clearly prove its pathogenicity. Exogenous expression of p.G337E AFG3L2 in an *Afg3l2* null background indicates that the mutant protein has no residual function. Indeed, the complete loss of L-OPA1 and mitochondrial fragmentation in this condition were comparable to those previously observed in *Afg3l2* null MEFs [10]. In agreement, we found swollen mitochondria with altered cristae in the optic nerve of *Afg3l2* null mice (*VB and FM unpublished observation*). Investigations in patient fibroblasts also revealed faster abnormal processing of OPA1 despite the heterozygous state of the mutation, with strong reduction of L-OPA1, accumulation of S-OPA1 and altered mitochondrial fusion. Interestingly, the decrease in L-OPA1 is significantly more pronounced in the proband compared to his affected mother, in line with the more severe phenotype, indicating that OPA1 processing might be considered as an outcome of disease severity in this form of ADOA. We also demonstrated that OPA1 processing is caused by strong OMA1 hyper-activation, which is reduced in amount in patients versus controls because of its faster autocatalysis. On the contrary, YME1L1 levels were not altered in patients, thus excluding a compensatory upregulation of YME1L1 on the final outcome on mitochondrial dynamics.

OPA1 processing is more severely compromised in this family compared to what we previously described in SCA28 and SPAX5-patient fibroblasts, where the levels of L-OPA1 were moderately reduced compared to controls, the accumulation of S-OPA1 was not appreciated, and the mitochondrial network presented shorter tubules, but not evident fragmentation [7]. Interestingly, the mutation we identified localizes close to the AAA domain, while most of those associated with SCA28 or SPAX5 affect the proteolytic domain, suggesting that mutations in different domains of this protein could differently affect its molecular function. Mutations localizing in the AAA domain of AFG3L2 can abolish ATP binding/hydrolysis and impact more severely on proteolytic activity, in agreement with a recent work in which ATPase and proteolytic activity of AFG3L2 carrying different mutations were assessed in vitro [19].

SCA28 and SPAX5 predominantly affect the cerebellum, while this novel *AFG3L2* mutation predominantly affects the optic nerve. The aberrant OPA1 processing and severe mitochondrial fragmentation we observed, together with the fact that most ADOA patients carry OPA1 mutations, indicates that a fine control of mitochondrial dynamics is crucial for RGC survival. We may speculate that *AFG3L2* mutations that predominantly and severely impact on OPA1 processing affect specifically RGCs, while those mostly impinging on other AFG3L2-related functions (oxidative phosphorylation and mitochondrial calcium homeostasis) affect Purkinje neurons in the cerebellum [10, 20, 21]. Purkinje neurons are selectively vulnerable to these defects, since they are characterized by a high oxidative metabolism and experience elevated calcium fluxes due to massive glutamatergic stimulations [22, 23].

In conclusion, our study broadens the spectrum of neurodegenerative diseases associated with *AFG3L2* mutations and expands the genetic causes leading to ADOA, enforcing aberrant OPA1 processing as common mechanism for this disease.

## Data Availability

All data generated or analyzed during this study are included in this article.

## **Abbreviations**

AAA: ATPase associated with a variety of cellular activities
ADOA: Autosomal dominant optic atrophy
CSF: Cerebrospinal fluid
IMM: Inner mitochondrial membrane
L-OPA: Long OPA1 forms
MEF: Murine embryonic fibroblast
MRI: Magnetic resonance imaging
MS: Multiple sclerosis
NMO-IgG: Neuromyelitis optica-Immunoglobulin G
RGC: Retinal ganglion cell
SCA28: Spinocerebellar ataxia type 28
SPAX5: Spastic ataxia 5
S-OPA1: Short OPA1
WB: Western blot
WT: Wild type
YFP: Yellow fluorescent protein
